# Supplementary material for: Fast rotating blue stragglers prefer loose clusters
Source: Nat Commun. 2023 May 15;14:2584. doi: 10.1038/s41467-023-38153-w (PMC10185567; doi:10.1038/s41467-023-38153-w)
Supplement: Supplementary file 1 — Supplementary Information file [file 41467_2023_38153_MOESM1_ESM.pdf]

Supplementary Information for
“Fast rotating Blue Stragglers prefer loose clusters”

Francesco R. Ferraro[1,2*], Alessio Mucciarelli[1,2], Barbara Lanzoni[1,2], Cristina Pallanca[1,2], Mario Cadelano[1,2], Alex Billi[1,2], Alison Sills[3], Enrico Vesperini[4], Emanuele Dalessandro[2], Giacomo Beccari[5], Lorenzo Monaco[6], Mario Mateo[7]

[1]Dipartimento di Fisica e Astronomia “Augusto Righi”, Alma Mater Studiorum Universita` di Bologna, Via Piero Gobetti 93/2, I-40129 Bologna, Italy
[2]INAF -- Astrophysics and Space Science Observatory Bologna, Via Piero Gobetti 93/3, I-40129 Bologna, Italy
[3]Department of Physics & Astronomy, McMaster University, 1280 Main Street West, Hamilton ON, L8S 4M1, CANADA
[4]Department of Astronomy, Indiana University, Bloomington, IN, 47401, USA
[5]European Southern Observatory, Karl-Schwarzschild-Strasse 2, 85748 Garching bei Munchen, Germany
[6]Departamento de Ciencias Fisicas, Universidad Andres Bello, Fernandez Concha 700, Las Condes, Santiago, Chile
[7]Department of Astronomy, University of Michigan, 1085 S. University, Ann Arbor, MI 48109,USA

The Data-set: For this study we have used a collection of high-resolution spectra acquired over the last 15 years (see Supplementary Table 2). Most of the data have been acquired with the multi-object spectrograph FLAMES-GIRAFFE[1] mounted at the Very Large Telescope (VLT) of the European Southern Observatory (ESO). The instrument allows the simultaneous acquisition of high resolution (R=10000-30000) spectra for about 130 targets over a field of view of 25’ in diameter. Part of the dataset has been already published and we refer to the original papers for the detailed description of the observations and data reduction: 47 Tucanae[2], M4[3], NGC 6397[4], M30[5], NGC 6752[6], ω Centauri[7]. For M55 the datasets have been acquired with FLAMES@VLT under program 093.D-0270 (PI: Lovisi), in June-July 2014, through 16 repeated exposures (each one of ~ 2760 sec) in the spectral region 3854-4049 Å, with a spectral resolution of 19,600.

In the case of NGC 3201 we used the multi-object fiber system Michigan/Magellan Fiber System (M2FS)[8], which feeds the double spectrograph MSPec mounted at the Magellan Clay Telescope at the Las Campanas Observatory, in Chile. This instrument allows the simultaneous observation of up to 128 objects per spectrograph over a field of view of about 30’ of diameter. The data have been acquired in February-March 2019, through 16 repeated exposures in the spectral region 5127-5184 Å, with a spectral resolution of 18000, thus allowing us to properly sample the Mg lines at 5167.3 Å and 5172.6 Å.

The spectroscopic sample- The spectroscopic targets in each cluster have been selected to provide a reasonable sampling of the BSS population in both luminosity and radial distribution. The selection is based on extensive photometric catalogues (in most cases covering the entire cluster extension) published by our group over the years for each investigated system: ωCentauri[9], M30[10], 47Tucanae[11], NGC6752[12], M55[13], M4[3]. Typically, we used ultraviolet Hubble Space Telescope data in the central region (thus minimizing crowding issues) and ground-based optical observations for the outskirts. Of course, to keep a reasonably high signal-to-noise level, the faint extension of the BSS sequence is in general not included in the sample. In addition, to avoid possible biases due to effects of internal dynamical evolution, special care was devoted to properly sample the entire BSS radial distribution in each cluster, including the innermost regions where mass segregation processes and stellar interactions are particularly efficient. To avoid contamination of the spectra from scattered light, only isolated BSSs have been selected: all the BSSs having stars of comparable or brighter luminosity within a distance of about 3” (which is more than twice the fiber size) have been excluded from the selection. With these constraints, we succeed in safely allocating some fiber even in the central regions of high-density clusters. For instance, half of the spectroscopic sample (7 BSSs out of 15) has been observed within the innermost 15” from the centre of M30, which is a very concentrated, post-core collapse system. The number of surveyed BSSs is provided in Supplementary Table 1 and their distribution on the plane of the sky is plotted in Supplementary Figure 1, which clearly shows that, in all cases, the observed samples probe the central part of the cluster and extend to the external regions (out to 1-2 half-mass radii).

Supplementary Table. 1 | The BSS sample and the main parameters of the target clusters.

**Name**	**N_{TOT}**	**N_{FR}**	**[Fe/H]**	**M_V**	**c**	**log ρ_0**	**Γ_{coll}**	**A^+**	**Reference**
M55	32	15	-1.9	-7.57	0.93	2.22	5.4×10^4	0.10	This paper
NGC3201	67	19	-1.6	-7.45	1.29	2.71	1.0×10^5	0.19	This paper
ω Centauri	109	45	-1.6	-10.26	1.31	3.15	1.9×10^6	0.00	Mucciarelli et al. (2013)
M4	20	8	-1.2	-7.19	1.65	3.64	4.5×10^5	0.12	Lovisi et al. (2010)
47 Tucanae	43	1	-0.7	-9.42	2.1	4.88	1.4×10^7	0.29	Ferraro et al. 2006
M30	15	1	-2.2	-7.45	2.5	5.01	2.2×10^6	0.52	Lovisi et al. (2013a)
NGC6752	18	0	-1.5	-7.73	2.5	5.04	4.7×10^6	0.33	Lovisi et al. (2013b)
NGC6397	16	2	-2.0	-6.64	2.5	5.76	1.5×10^6	0.69	Lovisi et al. (2012)

The numbers of observed BSSs (N_{TOT}) and fast rotators (N_{FR}) are listed in columns 2 and 3, respectively, and the corresponding reference publication is reported in column 9. The values of iron abundance ([Fe/H]), integrated absolute magnitude (M_V), King concentration parameter (c), and central luminosity density (log ρ_0) are taken from the Harris Globular Cluster Catalog[14], those of the collisional parameter Γ_{coll} have been computed as described in Methods, and those of the A^+ parameter are from the literature[15].

Supplementary Table 2 | The observational data base

CLUSTER	PROGRAMME	PI	INTRUMENT	ONLINE DATA AVAILABILITY
47 Tucanae	072.D-0337	FERRARO	ESO-VLT-FLAMES	1
ω Centauri	077.D-0696<br>081.D-0356<br>089.D-0298	FREYHAMMER<br>FERRARO<br>FERRARO	ESO-VLT- FLAMES<br>ESO-VLT- FLAMES<br>ESO-VLT- FLAMES	1
M4	081.D-0356	FERRARO	ESO-VLT- FLAMES	1
M30	087.D-0748<br>089.D-0306	LOVISI<br>FERRARO	ESO-VLT-FLAMES<br>ESO-VLT-X-SHOOTER	1
NGC 6397	073.D-0093<br>081.D-0356	FERRARO<br>FERRARO	ESO-VLT- FLAMES<br>ESO-VLT- FLAMES	1
NGC 6752	081.D-0356<br>089.D-0298	FERRARO<br>FERRARO	ESO-VLT- FLAMES<br>ESO-VLT- FLAMES	1
M55	093.D-0270	LOVISI	ESO-VLT- FLAMES	1
NGC 3201	CN2019A-15	MONACO	MAGELLAN-M2FS	2

1. http://archive.eso.org/eso/eso_archive_main.html
2. http://www.cosmic-lab.eu/Cosmic-Lab/BSS_rotation_spectra.html

Supplementary Table 3 | The main best-fit relations. Parameters and uncertainties of the best-fit relations shown as solid lines in the figures listed in the first column.

Figure	Best fit relations	rms
Figure 3a	$f_{FR} = -0.26\ (\pm 0.06) \times c + 0.72\ (\pm 0.12)$	0.09
Figure 3b, 4b	$f_{FR} = -0.12\ (\pm 0.03) \times \log(\rho_0) + 0.74\ (\pm 0.14)$	0.10
Figure 3c	$f_{FR} = -0.18(\pm 0.06) \times \log(\Gamma_{coll}) + 1.29\ (\pm 0.39)$	0.12
Figure 3d	$f_{FR} = -1.43(\pm 0.46) \times A^{+} + 0.52\ (\pm 0.08)$	0.08
Figure 4a	$f_{FR} = 3.21(\pm 0.97) \times f_{BIN} + 0.01\ (\pm 0.07)$	0.10
Figure 4b	$f_{BIN} = -0.03(\pm 0.01) \times \log(\rho_0) + 0.18\ (\pm 0.04)$	0.03

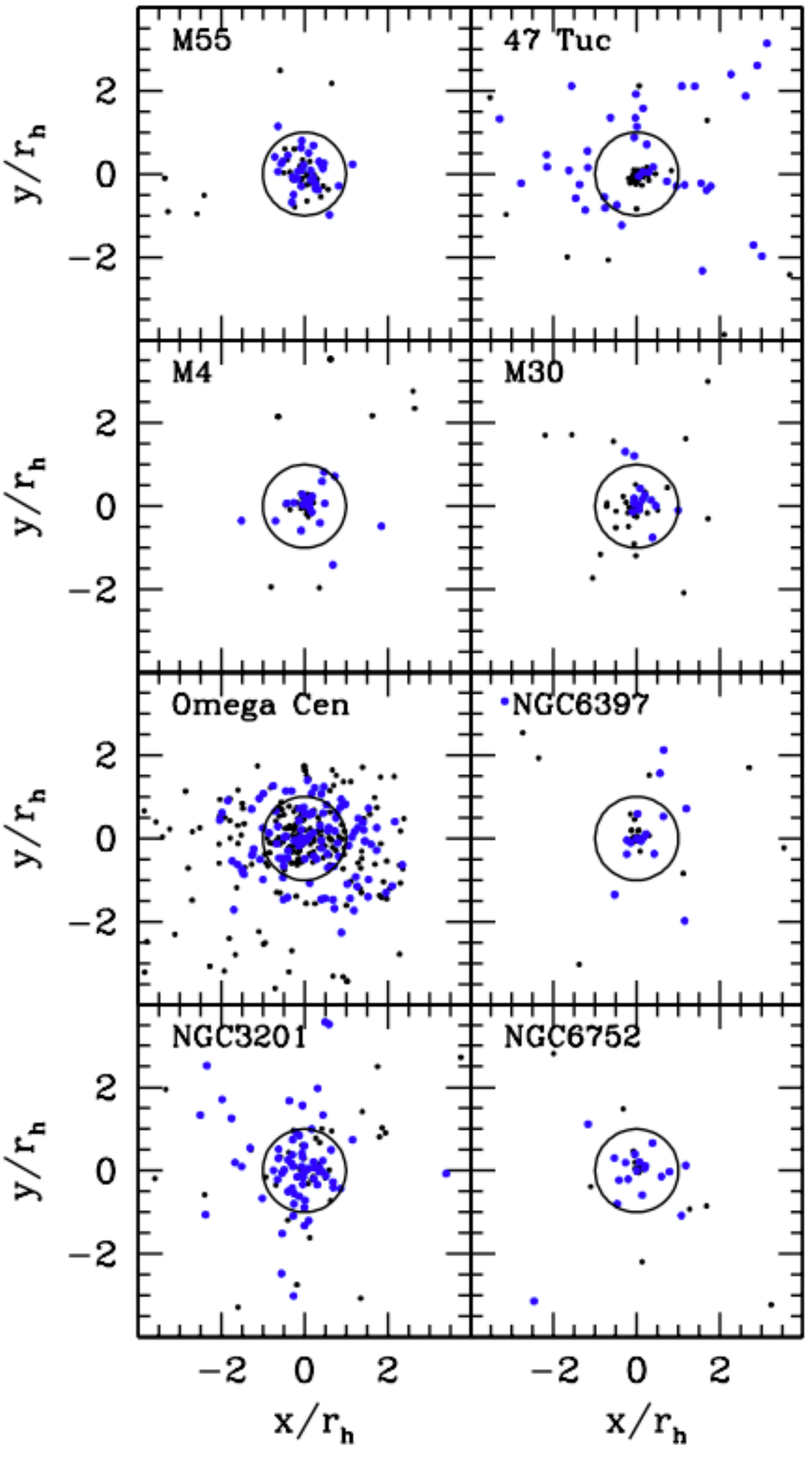

Supplementary Fig. 1 – The radial distribution of the spectroscopic sample. The sky distribution of the BSSs with measured rotation velocity (blue circles) is shown for each target cluster. The BSSs not observed in the spectroscopic survey are shown as black dots and are not included in the statistics. The x and y distances from the centre are plotted in units of the cluster half-mass radius (r_h). The black circles have radius equal to one r_h.

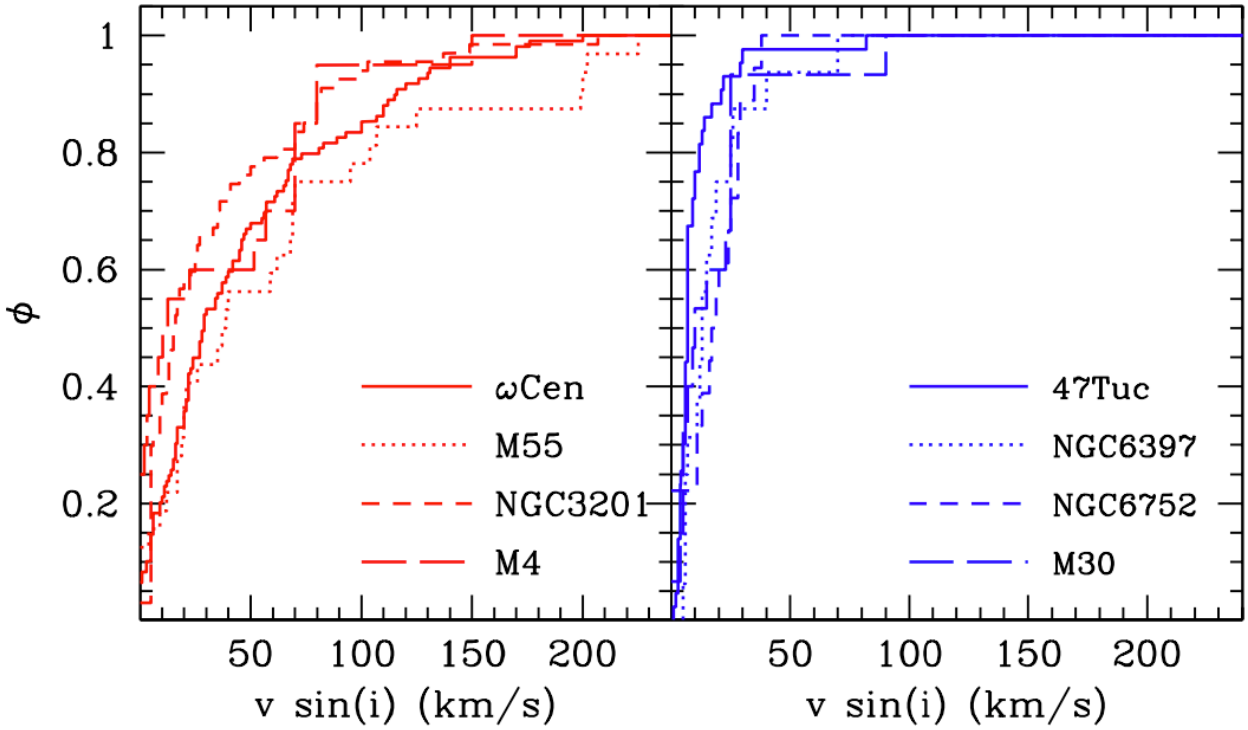

Supplementary Fig. 2 – Comparing the rotation velocity distributions. The normalized cumulative distributions (ϕ) of rotation velocities (v sin i) measured in the programme clusters. The four low-density clusters in the left panel (namely ωCentauri, M55, NGC 3201 and M4) show shallower cumulative distributions (indicating larger numbers of fast rotating BSSs), than the four high-density clusters in the right panel (namely 47 Tucanae, NGC 6397, NGC 6752 and M30).

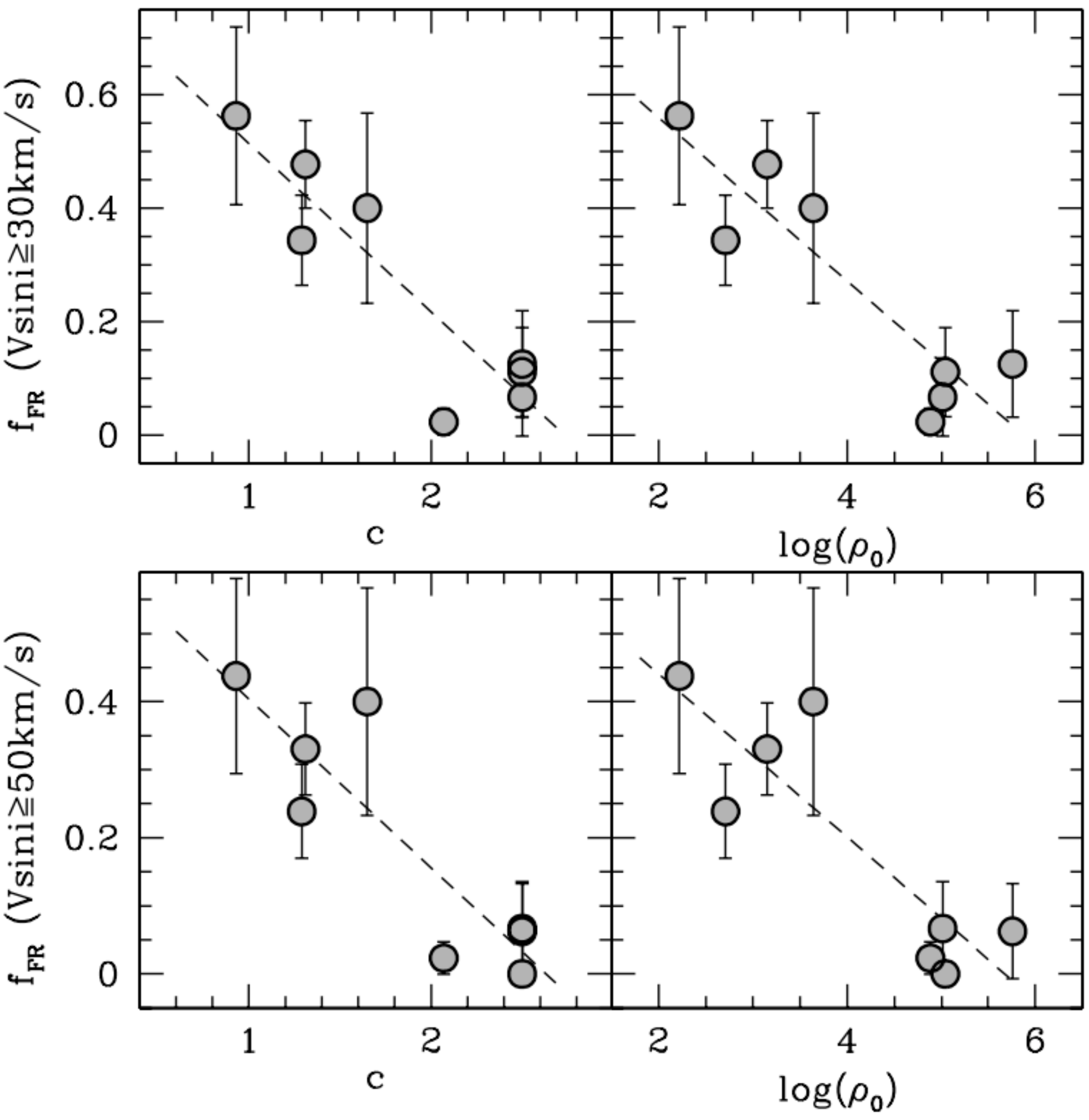

Supplementary Fig. 3 – Changing the FR threshold. Fraction of FRs, f_{FR}(grey circles) as a function of the cluster concentration ($c=\log(r_t/r_c)$, where r_t is the tidal radius and r_c is the core radius of the cluster, left panels) and central luminosity density ($\log \rho_0$ in units of $L_\odot/pc^3$; right panels) for two different assumptions of the threshold used to define FRs: 50 km/s (top panels) and 30 km/s (bottom panels). The comparison between this figure and Figure 3 clearly shows that the overall characteristics of the distribution remain unchanged independently of the adopted threshold. The errors are computed following the Poisson statistics. The dashed lines are the linear best fits to the data.

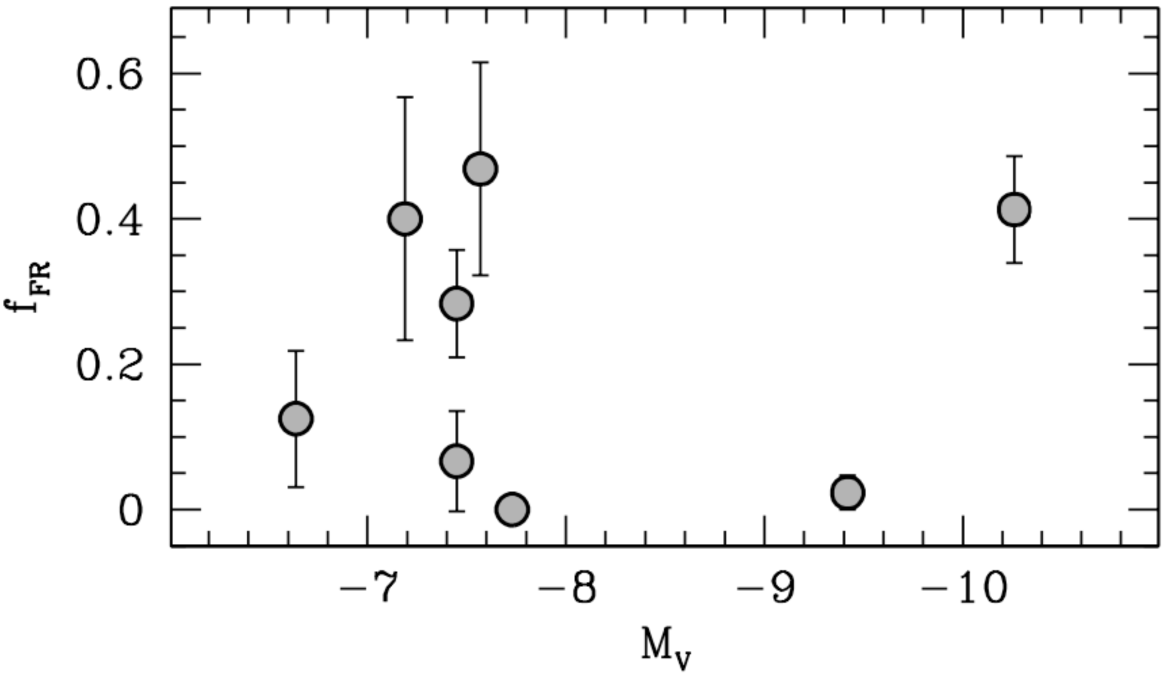

Supplementary Fig. 4 – An orthogonal information. While the overall fraction of BSSs is found[16-18] to decrease for increasing cluster luminosity, the fraction of FRs shows no significant correlation with this property (M_V is the integrated absolute magnitude of the surveyed clusters). The errors are computed following the Poisson statistics.

Supplementary References

1. Pasquini, L., et al. Installation and commissioning of FLAMES, the VLT Multifibre Facility. *The messenger*, **110**, 1 (2002)
2. Ferraro, F.R. et al. Discovery of Carbon/Oxygen-depleted Blue Straggler Stars in 47 Tucanae: The Chemical Signature of a Mass Transfer Formation Process. *Astrophys. J.* 647, L53 (2006);
3. Lovisi, L., et al. Fast Rotating Blue Stragglers in the Globular Cluster M4. *Astrophys. J.* **719,** L121-L125 (2010).
4. Lovisi, L., et al. Chemical and Kinematical Properties of Blue Straggler Stars and Horizontal Branch Stars in NGC 6397. *Astrophys. J.* **754,** 91 (2012).
5. Lovisi, L., et al. Flames and XSHOOTER Spectroscopy along the Two Blue Straggler Star Sequences of M30. *Astrophys. J.* **772,** 148 (2013).
6. Lovisi, L., et al. Another Brick in Understanding Chemical and Kinematical Properties of BSSs: NGC 6752. *Astrophys. J.* **778,** 64 (2013).
7. Mucciarelli, A., et al. Spinning Like a Blue Straggler: The Population of Fast Rotating Blue Straggler Stars in ω Centauri . *Astrophys. J.* **797,** 43 (2014).
8. Mateo et al. M2FS: the Michigan/Magellan Fiber System. *Proceedings of the SPIE*, **8446**, 84464Y (2012)
9. Ferraro F.R., et al. The Pure Noncollisional Blue Straggler Population in the Giant Stellar System ω Centauri. *Astrophys. J.* **638,** 433-439 (2006).
10. Ferraro, F.R. et al. Two distinct sequences of blue straggler stars in the globular cluster M 30. *Nature* **462,** 1028-1031 (2009).
11. Ferraro, F.R. et al. Discovery of Another Peculiar Radial Distribution of Blue Stragglers in Globular Clusters: The Case of 47 Tucanae. *Astrophys. J.* **603**, 127 (2004).
12. Sabbi, E., et al. The Small Blue Straggler Star Population in the Dense Galactic Globular Cluster NGC 6752. *Astrophys. J.* **617**, 1296 (2004).
13. Lanzoni, B. et al. The Surprising External Upturn of the Blue Straggler Radial Distribution in M55. *Astrophys. J.* **670**, 1065 (2007).
14. Harris, W.E. A catalog of parameters for globular clusters in the Milky Way. *Astron. J.* **112**, 1487-1488 (1996).
15. Ferraro, F. R., et al. The Hubble Space Telescope UV Legacy Survey of Galactic Globular Clusters. XV. The Dynamical Clock: Reading Cluster Dynamical Evolution from the Segregation Level of Blue Straggler Stars. *Astrophys. J.* **860,** 36 (2018).
16. Piotto, G., et al. Relative Frequencies of Blue Stragglers in Galactic Globular Clusters: Constraints for the Formation Mechanisms. *Astrophys. J.* **604,** L109 (2004).

17. Leigh, N. et al. Where the blue stragglers roam: searching for a link between formation and environment. *Astrophys. J.* **661,** 210 (2007).
18. Moretti, A., et al. Environmental effects on the globular cluster blue straggler population: a statistical approach. *Astronomy & Astrophysics*, **483**, 183 (2008)
